# Knowledge, Attitude, and Practices of Dental Surgeons in managing Child Patients

**DOI:** 10.5005/jp-journals-10005-1393

**Published:** 2016-12-05

**Authors:** Aisha Wali, Talha Mufeed Siddiqui, Rabia Khan, Kanza Batool

**Affiliations:** 1 Senior Lecturer, Department of Operative Dentistry, Baqai Dental College Karachi, Sindh, Pakistan; 2 Associate Professor, Department of Operative Dentistry, Baqai Dental College Karachi, Sindh, Pakistan; 3 Demostrator, Department of Oral Biology, Baqai Dental College, Karachi Sindh, Pakistan; 4 House Surgeon, Department of Operative Dentistry, Baqai Dental College Karachi, Sindh, Pakistan

**Keywords:** Attitude and practices, Behavioral techniques, Pediatric patients.

## Abstract

The aim of the study was to evaluate the knowledge, attitude, and practices of dental surgeons in the city of Karachi providing treatment to pediatric patients. A cross-sectional study was conducted to evaluate the knowledge, attitude, and practices of dental surgeons in the city of Karachi providing treatment to pediatric patients. A cluster-sampling technique was used and 200 dental surgeons from six different dental institutions were selected. A self-constructed questionnaire was distributed to the dental surgeons that comprised 20 closed-ended questions. The data was entered and analyzed for frequency and percentages by using Statistical Package for the Social Sciences (SPSS) version 19. The results showed that 76 (38%) dental surgeons took the responsibility of managing pediatric patient when given; 68 (34%) dental surgeons allowed the parents in the clinic; 111 (55.5%) dental surgeons are of the view that colorful and fun environment in dental clinic make the child at ease; 59 (29.5%) always demonstrate the dental procedure to the child to eradicate imaginary fears; 94 (47.0%) dental surgeons preferred the child to be treated in general anesthesia (GA) to avoid difficult behavior of the child; 135 (67.5%) dental surgeons did not show syringe needle or any instrument to the child. All the members of dental profession must be aware of patient perceptions, preferences, and fear to meet patient’s needs. Dental studies should include guidelines and techniques to train the upcoming dentists for excellent practice in pediatric dentistry.

**How to cite this article:**

Wali A, Siddiqui TM, Khan R, Batool K. Knowledge, Attitude, and Practices of Dental Surgeons in managing Child Patients. Int J Clin Pediatr Dent 2016;9(4):372-378.

## INTRODUCTION

Dental surgeons are expected to diagnose and manage effectively childhood dental diseases that are within the knowledge and skills acquired during dental education.^1^ Safe and effective treatment provided often requires modifying the child’s behavior.^2^ Pediatric dentistry is considered to be the most needed and yet neglected area of all the services performed by the dental surgeons.^3^ The auxiliary staff, as well as the clinical team, should be welcoming and friendly.^4^ Communication with the children should be age-specific, and the dental team should develop a specialized vocabulary.^5^ A child’s future attitude toward dentistry may be determined by a series of successful experiences in a pleasant dental environment. Dental surgeons should be encouraged to increase and update their clinical skills and knowledge in behavior guidance techniques by reading dental literature, observing video presentations, or attending continuing education programs.^6^ The establishment of good relationship between dentist and the child has been shown to increase the success of treatment in terms of the child’s cooperation during the treatment or advice for preven-tion.^7^ Parents exert a significant influence on their child’s behavior, especially if they had previous negative dental experiences.^8-10^ An anxious or fearful parent may affect the child’s behavior negatively.^8,9,11^ Educating the parent before the child’s first appointment is important, and effective communication with more demanding parents represents an opportunity for the dental surgeon to carefully review behavior and treatment options and together decide what is in the child’s best interests.^12^ Dental surgeons have the same opinion that good communication is important amongst the dentist, patient, and parent in building trust and confidence.^12,13^ Communication skills of the dental surgeons play an important role in behavior guidance and the health professionals may be inattentive to communication style, but parents/patients are very attentive to it.^14^ Dental surgeons behaviors reported to correlate with low parent satisfaction include rushing through appointments, not taking time to explain the procedures, barring parents from the examination room, and generally being impatient.^15^ Dental surgeons behavior of vocalizing, directing, empathizing, persuading, giving the patient a feeling of control, and operant conditioning have been reported as efficacious responses to uncooperative patient behaviors.^16^ The most common emotional upsets seen during dental treatment are anxiety and fear, which might originate from a previous traumatic experience in the dental office or during hospitalization for other reasons.^17^ Dental anxiety and fear of dental treatment in children are considered to be the main reason for management problems and avoidance of dental care. These problems sometimes require replacement of conventional treatment with more complicated alternatives, such as sedation or general anesthesia (GA).^18^ Children who have positive interactions with their dentist will be more likely to visit the dentist and will have better dental health.^19^ Moreover, pediatric dentistry demands the use of diagnostic aids as well as correct interpretation of findings both in emergency and in routine problems.^20^ Various barriers including developmental delay, physical/ mental disability, and acute or chronic disease all are potential reasons for noncompliance and may hinder the achievement of a successful outcome. To alleviate these barriers, the dental surgeon should become a teacher and the methods should include active listening and observation of child’s body language.^21^ Shortcoming of most of the dental surgeons when treating children is their lack of knowledge, clinical skill, or attention to the vital performance of providing and assuring profound local anesthesia. Most of the dental surgeons felt uncomfortable with their clinical skills and avoid giving children local anesthesia. For this vision to become reality, many more dental professionals will need to be aware of and skilled in the communication management methods advocated by the American Academy of Pediatric Dentistry.^22^

Therefore, the aim of the study was to evaluate the knowledge, attitude, and practices of dental surgeons in the city of Karachi providing treatment to pediatric patients.

## MATERIALS AND METHODS

### Study Design

A cross-sectional study was conducted in May 2014 to February 2015 to evaluate the knowledge, attitude, and practices of dental surgeons in the city of Karachi providing treatment to pediatric patients.

### Ethical Approval

The study was approved by the Ethical Committee, Baqai Medical University.

### Sampling Technique

**Fig. 1:**
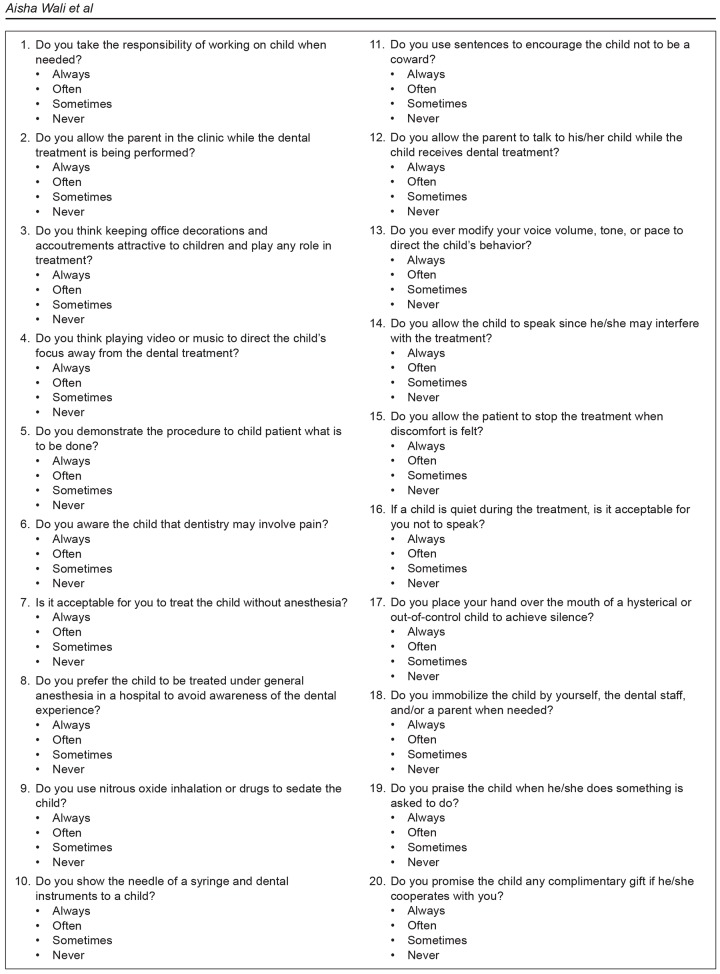
Questionnaire

A cluster-sampling technique was used and 200 dental surgeons from six different dental institutions were selected. A self-constructed questionnaire (Fig. 1) was distributed to the dental surgeons that comprised 20 closed-ended questions, including the parental influence, communication with the child, decorations and accouterments depicting definite settings, importance of demonstrating a child about treatment, sedative procedures, and various barriers that hinder the dental treatment.

Inclusion Criteria

Dental surgeons with the clinical experience of 3 years and above currently working in dental institutes of Karachi.

Exclusion Criteria

Clinical experience below 3 years. Currently not working in a dental institution.

### Statistical Analysis

The data was entered and analyzed for frequency and percentages by using Statistical Package for the Social Sciences (SPSS) version 19.0.

## RESULTS

The present study comprised 200 dental surgeons of experience level 3 years and above. Table 1 shows the descriptive analysis of the knowledge, attitude, and practices of dental surgeons providing treatment to pediatric patient. Results showed that 76 (38%) dental surgeons took the responsibility of managing pediatric patient when given; 68 (34%) dental surgeons allowed the parents in the clinic as a spectator to encourage and assure the child to work in a satisfactory manner; 111 (55.5%) dental surgeons are of the view that colorful and fun environment in dental clinic makes the child at ease, while 67 (33.5%) dental surgeons think that having a handy music system/video will provide comfort to frightened children; 59 (29.5%) always demonstrate the dental procedure to the child to eradicate imaginary fears; 102 (51%) preferred not to inform the child that the dental procedure could involve pain. Table 2 shows behavior attributes of dentists toward pediatric patients; 109 (54.5%) dental surgeons preferred to treat the child without anesthesia to prevent from unpredictable behavior of child; 94 (47.0%) dental surgeons preferred the child to be treated in GA to avoid difficult behavior of the child; 135 (67.5%) dental surgeons did not show syringe needle or any instrument to the child as a good policy to carry out the treatment; 105 (52.5%) praised the good behavior of child to acknowledge exemplary conduct in a child. Table 3 shows sedation techniques used by dental surgeons on pediatric patient. Of dental surgeons, 76 (38.0%) occasionally allow the parents to take part in treatment verbally to approach the psychological management of the patient; 61 (30.5%) tended to modify their voice, tone to direct child’s behavior; 80 (40%) gave the child an opportunity to participate in the procedures; 94 (47.0%) of dental surgeons deferred the treatment when discomfort is felt; 69 (34.5%) dental surgeons chose not to engage the child in a conversation if he is not willing or showing interest; 136 (68%) dental surgeons never placed their hand on the mouth of a screaming spoiled child; 90 (45%) dental surgeons immobilize the child by their self and avoid any auxiliary help; 116 (58%) dentists praise the child if he obeys a command in a determined way; 58 (29%) dental surgeons promised to gift or reward a child to attain his maximum cooperation. Table 4 shows treatment modalities by different dental surgeons.

**Table 1:** **Table 1:** Descriptive analysis of the knowledge, attitude, and practices of dental surgeons providing treatment to pediatric patients

*Questionnaire*		*Mean*		*Standard* *deviation*	
Responsibility of managing child patient		2.09		0.939	
Allow parent in clinic		2.17		1.020	
Keep office decor		1.65		0.838	
Talking and playing video or music to		2.24		1.085	
distract the child					
Demonstrate procedure		2.32		1.078	
Aware child of pain		3.14		1.059	
Treat without anesthesia		3.34		0.841	
Prefer GA for treatment		3.34		0.816	
Nitrous oxide inhalation to sedate		3.06		0.671	
Don’t show needle of syringe		3.46		0.907	
Encourage child not to be coward		1.85		1.060	
Allow parent to interrupt		2.46		1.031	
Modify voice, tone to direct child behavior		2.22		1.018	
Allow child to speak		2.48		0.997	
Patient to stop treatment on discomfort		1.93		1.010	
Stop hysterical child by hand on mouth		3.49		0.845	
Immobilize the child		2.91		0.920	
Praise the child		1.68		0.934	
Give complimentary gifts		2.46		2.288	

**Table 2:** **Table 2:** Behavior attributes of dentists toward pediatric patients

		*Frequency/percentages*							
*Questionnaire*		*Always*		*Often*		*Sometimes*		*Never*	
Dental surgeons that manage pediatric patients		73(36.5%)		44(22%)		76(38%)		7(3.5%)	
Dental surgeons that allow parents in dental clinic		68(34%)		50(25%)		61(30.5%)		21(10.5%)	
Dental surgeons keep off decor attractive		111(55.5%)		56(28%)		26(13%)		7(3.5%)	
Talking and playing video or music to distract the child		67(33.5%)		50(25%)		51(25.5%)		32(16%)	
Dental surgeons who demonstrate the procedure to child		59(29.5%)		54(27%)		52(26%)		35(17.5%)	
Dental surgeons who encourages child not to be coward		105(52.5%)		44(22%)		27(13.5%)		24(12%)	

**Table 3:** **Table 3:** Sedation techniques used by dental surgeons on pediatric patient

		*Frequency and percentages*							
*Questionnaire*		*Always*		*Often*		*Sometimes*		*Never*	
Who aware the child about involvement of pain		25 (12.5%)		25 (12.5%)		48 (24%)		102 (51%)	
Who treat the child without anesthesia		7 (3.5%)		27 (13.5%)		57 (28.5%)		109 (54.5%)	
Who prefer the child to be treated in GA		9 (4.5%)		34 (17%)		94 (47%)		63 (31.5%)	
Who use nitrous oxide inhalation to sedate the child		14 (7.0%)		15 (7.5%)		36 (18%)		140 (70%)	
Who do not show needle of syringe/instrument to child		14 (7.0%)		15 (7.5%)		36 (18%)		135 (67.5%)	

**Table 4:** **Table 4:** Different treatment modalities by dental surgeons

		*Frequency and percentages*							
*Questionnaire*		*Always*		*Often*		*Sometimes*		*Never*	
Who modify their tone to direct child’s behavior		61(30.5%)		59(29.5%)		55(27.5%)		25(12.5%)	
Who allow child to speak during treatment		44(22%)		46(23%)		80(40%)		30(15%)	
Who stop treatment when discomfort is felt		94(47.0%)		41(20.5%)		50(25%)		15(7.5%)	
Who do not speak if child is quiet		42(21%)		43(21.5%)		69(34.5%)		46(23%)	
Who place hand-over-mouth of hysterical child		7(3.5%)		25(12.5%)		32(16%)		136(68%)	
Who immobilize the child by themselves		20(10%)		34(17%)		90(45%)		56(28%)	
Who praise child when obeys command		116(58%)		45(22%)		26(13%)		13(6.5%)	
Who promise complimentary gifts		58(29.0%)		52(26%)		57(28.5%)		32(16%)	

## DISCUSSION

Results showed that 76 (38%) of the dental surgeons took the responsibility of managing pediatric patients in dental clinics. The present survey done was one of the first kind to assess the behavior and attitudes of dental surgeons in Pakistan toward managing and treating pediatric patients. The findings from this survey that 38% of the dental surgeons provide treatment to children is not encouraging. Dental surgeons may simply be reluctant to see children so young because they perceive them to be difficult to examine. Further, they may not know what to do if, during the examination, it is discovered that the child will require further treatment. Educational programs should be planned for dental surgeons to improve their knowledge and skills in providing treatment to children.^22^ A survey done in Saudi Arabia reported that 85% of the dental surgeons treat children which is encouraging.^23^ Another study conducted by Seale and Casamassimo^24^ reported that more than 90% of dental surgeons provide treatment to children younger than 4 years of age.

Pain management during dental procedures is essential for successful behavior guidance and enhancing positive dental attitudes for future appointments. Listening to the child and observing their behavior at first sign of distress would help in diagnosing the situation and facilitate proper behavior guidance techniques.^25^ Children perceive and react to painful stimuli differently from each other and under the age of 4 years are more sensitive to painful stimuli and are not able to communicate as well as older children and teens.^21,26^ Observing behavior and listening to children during treatment are essential in any evaluation of pain. Facial expressions, crying, complaining, and body movement are important diagnostic criteria.^25,27-30^ The present study reported that 25 (12.5%) dental surgeons developed trust and explained the child about the nature of pain perception during dental procedures.

Parental accompaniment can significantly affect the atmosphere surrounding the dental visit and dental treatment and may sometimes enhance and sometimes hinder the progress of the child’s treatment.^7^ The present study results showed that 68 (34%) of the dental surgeons allowed parents in dental clinic. Levy and Domoto^31^ reported that 88% of dental surgeons and auxiliary staff allowed parents in the dental clinics. A survey done by the Association of Pedodontic Diplomats,^32^ nearly 90% of the dental surgeons allowed parents in the dental clinic. Another study^33^ reported that 35% of general dentists and 87% of pediatric dental surgeons allowed parents in the operatory.

Behavior guidance is a clinical art form and a skill built on a foundation of science with the goals to establish communication, alleviate fear and anxiety, deliver quality dental care, build a trusting relationship between dentist, child, and parent, promote the child’s positive attitude to dental health.^2^ The most popular technique for managing children was tell-show-do and was reported by 213(93%) dental surgeons as their most commonly used behavioral management strategy followed by 149(69%) reported voice control. The technique dentists were least comfortable with was hand-over-mouth; 7(3%) dental surgeons reported feeling uncomfortable with hand-over mouth techniques, followed by 5(2%) with the papoose board.^34-36^ The present study results reported that 67(33.5%) of the dental surgeons used distraction technique followed by 61(30.5%) used voice control technique, 59(29.5%) tell-show-do technique, 20(10%) used papoose board, and 7(3.5%) used hand-over-mouth technique.

Dental surgeons make every effort to reduce or eliminate pain and anxiety experienced by children, but also to improve patient manageability and satisfaction.^37^ Klassen et al^38^ considered whether music could help control pediat-ric pain and anxiety. Filcheck et al^39^ found no differences in disruptive behaviors between music therapy and placebo overall, or by level of disruptiveness, there was a significant difference among the uncooperative children with respect to disruptive behaviors, crying and complaining, and physical restraint required. The present study reported that 67 (33.5%) dental surgeons play music/video to distract the child’s focus away from dental treatment.

Most children can be managed effectively using the techniques outlined in basic behavior guidance and these techniques should form the foundation for all of the management activities provided by the dental surgeon. The advanced behavior guidance techniques commonly used and taught in advanced pediatric dental training programs include protective stabilization, sedation, and GA.^18^ The sedation of children is different from the sedation of adults; sedation in children often is administered to control behavior to allow the safe completion of the dental procedure. A child’s ability to control his or her own behavior to cooperate for a procedure depends both on his or her chronologic and developmental age.^40^

Nitrous oxide (N_2_O) is an attractive agent for pediat-ric procedural sedation because it provides rapid onset and offset of sedation. Most research has used 50% N_2_O, and there have been concerns regarding the variability of the sedation provided.^41,42^ A study done by Sarah et al reported that only 12 (6%) of the dental surgeons preferred to use nitrous oxide as a behavorial management technique.^43^ Another study results reported that 159 (73%) of the dental surgeons were totally comfortable with nitrous oxide sedation technique.^7^ The present study results showed that 14 (7%) dental surgeons preferred to use nitrous oxide to sedate children.

Despite the risk of adverse events of GA, dental treatment performed in a hospital is generally considered safe.^44^ Pediatric dentists reported a favorable attitude toward dental treatment under GA, and many reported an increasing interest in utilizing this modality more frequently in their dental practices.^45^ Comprehensive dental care under GA is often more efficient and cost effective than repeated dental visits for restorative care utilizing other sedation methods.^46^ Dental restorations performed under GA, especially for the treatment of early childhood caries, are reported to have greater quality and durability than restorations placed under conscious sedation.^47,48^ Kain et al^49^ showed greater observed compliance during anesthetic induction. A study done by Manal et al reported that more than 50% of the general dentists and 60% of the pediatric dental surgeons reported the use of GA.^50^ In the survey by McKnight-Hanes et al,^35^ 60% of the pediatric dentists used GA in oral rehabilitation. It is likely that the differences are due to the fact that more than 60% of the dental surgeons were working in hospitals where facilities were usually provided for the utilization of GA. A study done by Crossley and Joshi^7^ reported that 98(45%) dental surgeons performed treatment under GA. The present study results showed that 9(4.5%) of the dental surgeons preferred the child to be treated in GA. Klingberg and Broberg^51^ reported that children and adolescents were expected to experience mild fear and anxiety during their dental treatment. Fear may be observed in children, adults, and the elderly, and it is suggested that young children and females are more likely to suffer from needle phobia.^52^ The present study results showed that 14(7%) of the dental surgeons showed needle to children during treatment.

## CONCLUSION

All the members of dental profession must be aware of patients’ perceptions, preferences, and fear to meet patient’s needs. Dental studies should include guidelines and techniques to train the upcoming dentists for excellent practice in pediatric dentistry.
